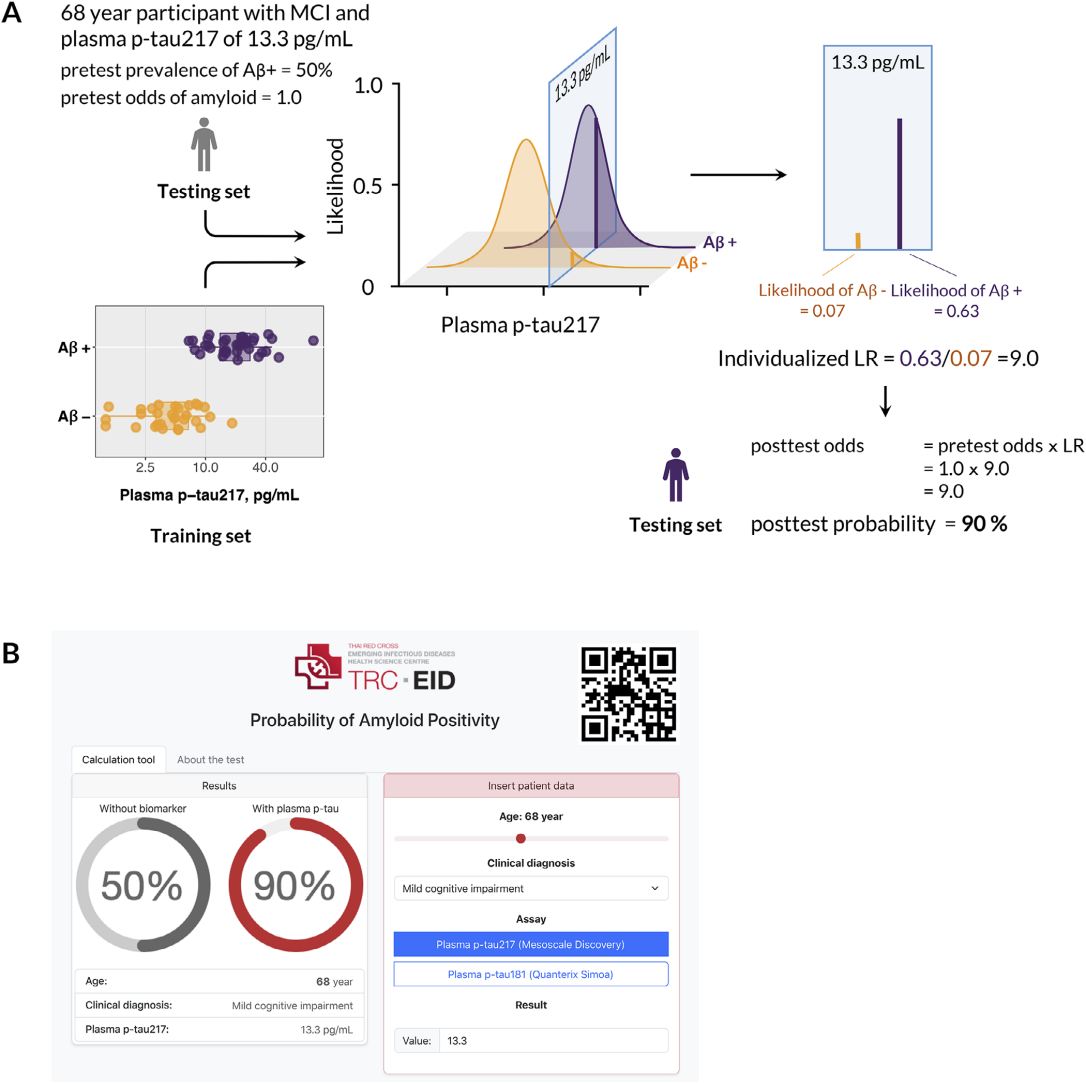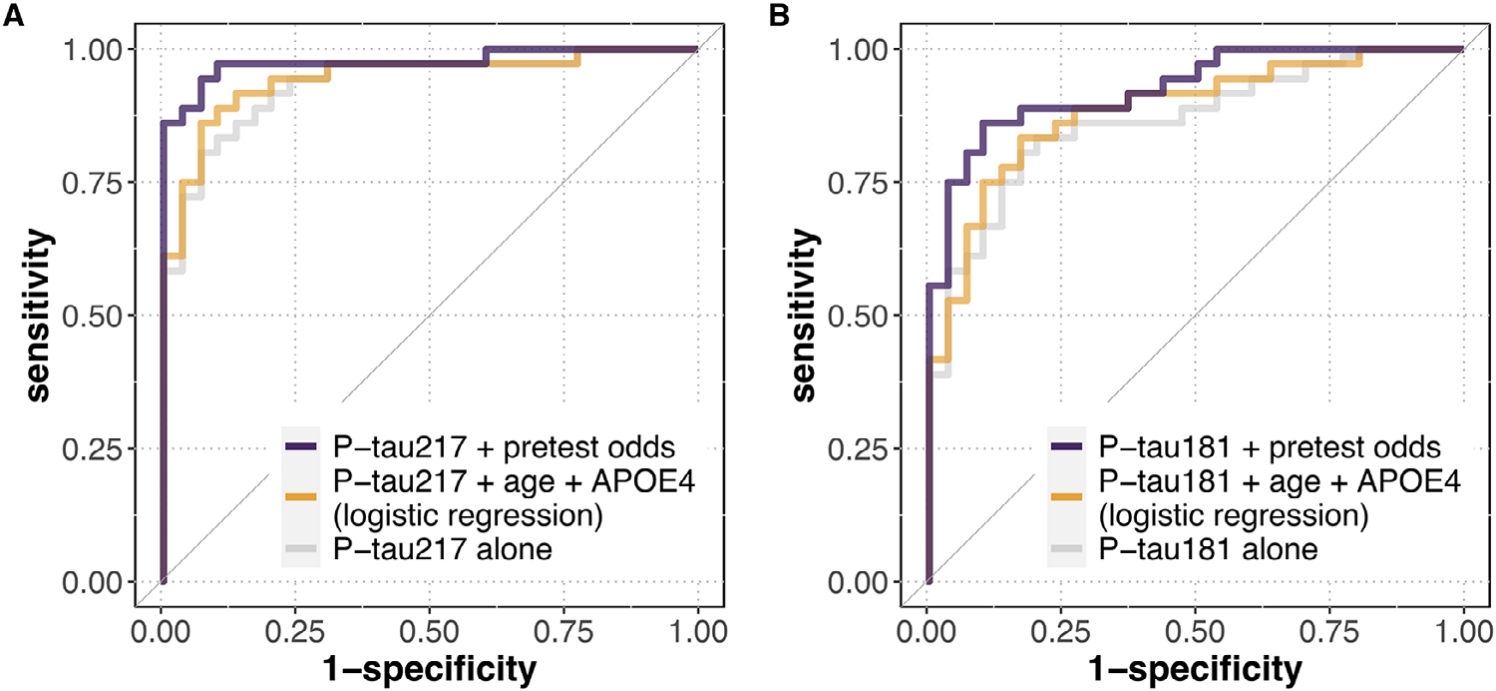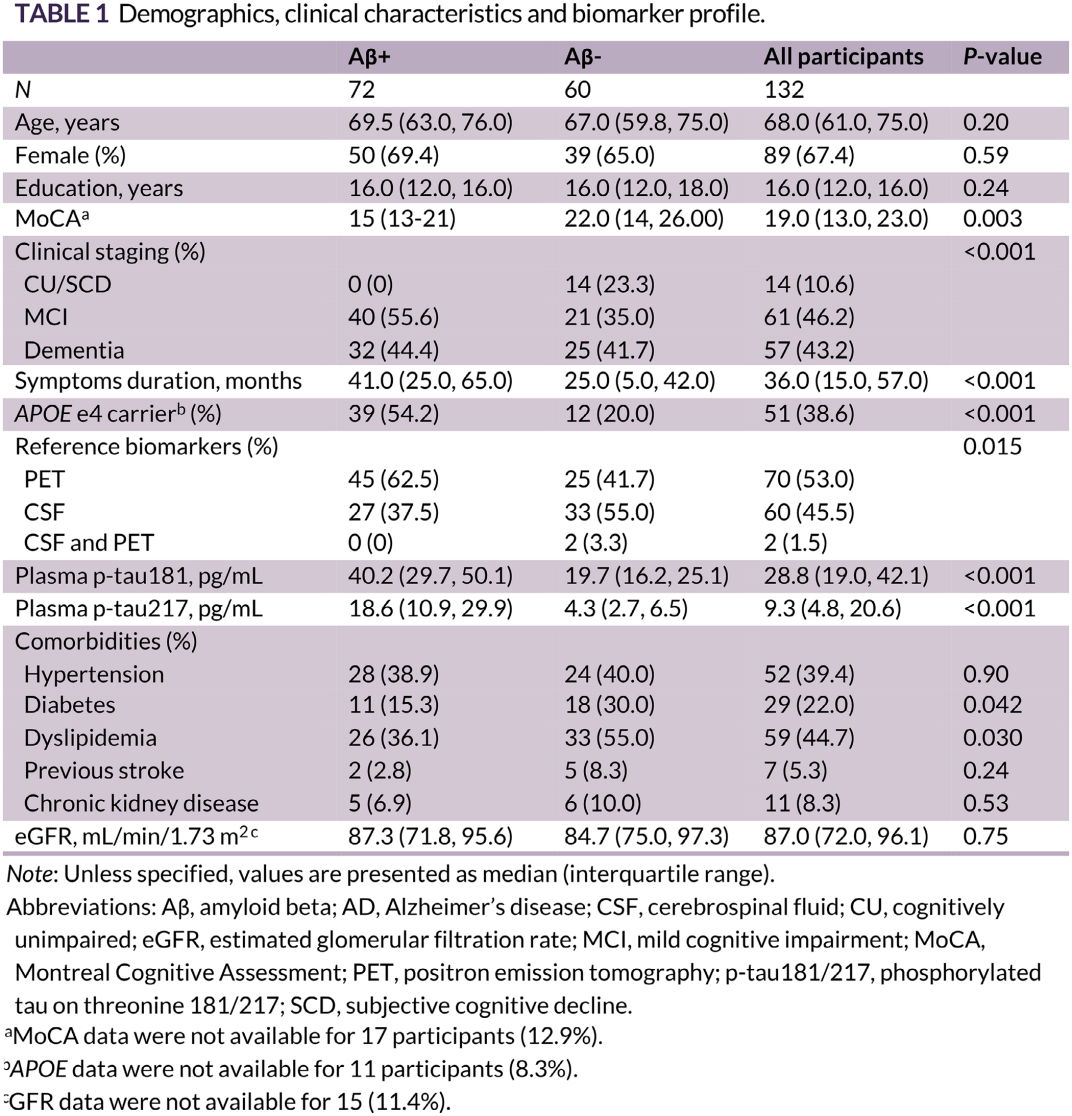# The real‐world implementation strategies for plasma p‐tau217 in tertiary care memory clinics in Thailand

**DOI:** 10.1002/alz.092457

**Published:** 2025-01-09

**Authors:** Poosanu Thanapornsangsuth, Kittithatch Booncharoen, Jedsada Khieukhajee, Watayuth Luechaipanit, Thanaporn Haethaisong, Adipa Chongsuksantikul, Thirawat Supharatpariyakorn, Chaipat Chunharas, Yuttachai Likitjaroen, Thiravat Hemachudha

**Affiliations:** ^1^ Thai Red Cross Emerging Infectious Diseases Health Science Centre, King Chulalongkorn Memorial Hospital, Bangkok Thailand; ^2^ Memory Clinic, King Chulalongkorn Memorial Hospital, Bangkok Thailand; ^3^ Division of Neurology, Department of Medicine, Faculty of Medicine, Chulalongkorn University, Bangkok Thailand; ^4^ Chula Neuroscience Center, King Chulalongkorn Memorial Hospital, Bangkok Thailand; ^5^ Neurology Center, Phyathai 1 Hospital, Bangkok Thailand; ^6^ Neurocognitive Unit, Division of Neurology, Faculty of Medicine, Chulalongkorn University, Bangkok Thailand; ^7^ Neurological Institute of Thailand, Ratchathewi, Bangkok Thailand; ^8^ Cognitive, Clinical and Computational Neuroscience (CCCN) Center of Excellence, Chulalongkorn University, Bangkok Thailand

## Abstract

**Background:**

Plasma tau phosphorylated at Thr217 (p‐tau217) has demonstrated excellent performance in identifying individuals with Alzheimer’s disease (AD) in both research and memory clinic settings. Nonetheless, implementing plasma p‐tau217 into clinical practice remains a challenge requiring careful clinical judgement and conservative interpretation near the cutpoint. The present study proposes a strategy that utilizes Bayes theorem in considering the clinical context, individualized (ie. non‐binary) likelihood ratios and validate it in a multicenter tertiary care memory clinic population in Bangkok, Thailand.

**Method:**

Patients from two memory clinics: King Chulalongkorn Memorial Hospital and the Prasat Neurological Institute who underwent CSF analysis for core AD biomarkers or amyloid PET (Florbetaben) were enrolled. Evaluation by neurologists specializing in neurocognitive disorders preceded biomarker testing, resulting in prespecified clinical diagnoses. Pretest odds of amyloid positivity in these subgroups were extrapolated from Jansen, 2022 and Ossenkoppele, 2015. Plasma p‐tau217 (MSD) and p‐tau181 (Simoa) were measured. Participants were randomly divided into training and testing sets. Two Gaussian distributions (Aβ+ and Aβ‐) were generated from plasma p‐tau values in the test set, enabling the calculation of individualized likelihood ratios which posttest odds for each participant were determined. This strategy was compared to multivariable logistic regression using p‐tau, age, and APOE4 as predictor variables. Cross‐validation (ratio= 50:50) was repeated for 5,000 iterations, and performance was assessed using ROC analysis.

**Result:**

Among 132 participants enrolled, 67.4% were female, and 43.2% had dementia. The median age was 68 years (interquartile range: 61, 75). AD was present in 54.5%, as determined by PET (54.5%) or CSF (46%). The strategy incorporating plasma p‐tau217 and pretest odds demonstrated an AUC of 0.979 (95% CI 0.958‐0.998), significantly outperforming logistic regression (AUC 0.948, p=0.0192) and p‐tau217 alone (AUC = 0.939, p=0.003). For plasma p‐tau181, the strategy showed an AUC of 0.926 (95% CI 0.876‐0.968), which is superior to p‐tau181 alone (AUC = 0.864, p=0.006) but not logistic regression (AUC 0.885, p=0.068).

**Conclusion:**

The proposed strategy in this study offers a flexible approach to address the limitations of plasma p‐tau in clinical practice, significantly outperforming multivariable logistic regression. An online calculation tool demonstrating its use is available at https://trceid.org/diagALZ/.